# How optimal foragers should respond to habitat changes: a reanalysis of the Marginal Value Theorem

**DOI:** 10.1007/s00285-013-0734-y

**Published:** 2013-10-26

**Authors:** Vincent Calcagno, Ludovic Mailleret, Éric Wajnberg, Frédéric Grognard

**Affiliations:** 1 INRA, UMR 1355 Institut Sophia Agrobiotech, Sophia Antipolis, France; 2 Université Nice Sophia Antipolis, UMR 1355 ISA, Sophia Antipolis, France; 3 CNRS, UMR 7254 ISA, Sophia Antipolis, France; 4 INRIA, BIOCORE, Sophia-Antipolis, France

**Keywords:** Behavior, Dispersal, Heterogeneity, Fragmented habitat, Patch, Sensitivity analysis, 92B05, 91C99, 35Q92

## Abstract

The Marginal Value Theorem (MVT) is a cornerstone of biological theory. It connects the quality and distribution of patches in a fragmented habitat to the optimal time an individual should spend exploiting them, and thus its optimal rate of movement. However, predictions regarding how habitat alterations should impact optimal strategies have remained elusive, with heavy reliance on graphical arguments. Here we derive the sensitivity of realized fitness and optimal residence times to general habitat attributes, for homogeneous and heterogeneous habitats, retaining the level of generality of the MVT. We provide new predictions on how altering travel times, patch qualities and/or relative abundances should affect optimal strategies, and study the consequences of habitat heterogeneity. We show that knowledge of average characteristics is in general not sufficient to predict the change in the average rate of movement. We apply our results to examine the conditions under which the optimal strategies are invariant to scaling. We prove a previously conjectured form of invariance in homogeneous habitats, but show that invariances to scaling are not generic in heterogeneous habitats. We also consider the relative exploitation of patches that differ in quality, clarifying the conditions under which it is adaptive to stay longer on poorer patches.

## Introduction

The Marginal Value Theorem (MVT) is an important and popular tenet of biological theory (Stephens and Krebs 1986), combining high generality and a relatively simple mathematical formulation. When resources are distributed as discrete patches throughout the habitat, the MVT predicts how long an individual should spend exploiting each patch before moving to another, depending on the kinetics of fitness accumulation within patches, and on the time it takes to move between patches (the travel time; Charnov 1976). This question has many applications in evolutionary biology, and beyond (Hayden et al. 2011; Rijnsdorp et al. 2011). The MVT for instance provides a framework to understand the optimal duration of copulation for males (Parker and Stuart 1976), the evolution of animal migration (Baker 1978), clutch-size (Wilson and Lessells 1994), foraging strategies across a broad range of taxa (Danchin et al. 2008), lysis time for bacteriolytic viruses (Bull et al. 2004), or the expected duration of interactions for cooperative cleaner fish (Bshary et al. 2008). In fragmented landscapes, the MVT gives a rationale to determine when individuals should start dispersing (Poethke and Hovestadt 2002), and yields quantitative predictions on the expected rate of movement throughout a habitat (Belisle 2005; Bowler and Benton 2005).

A key question is how optimal strategies should compare between patches or habitats that differ in quality (Stephens and Krebs 1986). However, this is not directly addressed by the MVT. Charnov’s 1976 seminal article established the existence of, and characterized, the optimal residence time on each patch, such that the long term average rate of gain, taken to be a predictor of fitness, is maximized. Yet, computing the optimal residence times requires specifying a specific functional form for the accumulation of gains in patches, and, even so, it is usually impossible to solve the equations analytically. This is at best feasible for some simple functions in homogeneous habitats (i.e. if all patches are identical; Stephens and Krebs 1986) or using tractable approximations (Parker and Stuart 1976; McNair 1982; Stephens and Dunbar 1993; Charnov and Parker 1995; Ranta et al. 1995). These difficulties seriously complicate the investigation of how optimal residence times vary with habitat characteristics (Sih 1980; Stephens and Krebs 1986; Charnov and Parker 1995). As an alternative, graphical methods have proven very intuitive and can accommodate arbitrary gain functions (Parker and Stuart 1976), so that even today most discussions of the MVT rely on graphical arguments (e.g. Danchin et al. 2008). But this is not without caveats. First, the graphical argument is restricted to homogeneous habitats, limiting the scope for predictions in heterogeneous habitats (Stephens and Krebs 1986). Second, the generality and robustness of conclusions is hard to assess, which has sustained some confusion in the literature. For instance, it is commonly claimed, and tested experimentally, that, under the MVT, residence times should be higher on better patches in a given habitat (e.g. Kelly 1990; Wajnberg et al. 2000), or that residence time should increase with patch quality (e.g. Riechert and Gillespie 1986; Astrom et al. 1990; Alonso et al. 1994; Tenhumberg et al. 2001; Corley et al. 2010; Rijnsdorp et al. 2011). However, theoretical investigations of different particular ways to alter patch quality have yielded variable predictions (Sih 1980; Charnov and Parker 1995; Ranta et al. 1995; Danchin et al. 2008). For example, from some simple gain functions, it has been argued that scaling the gain function vertically (a natural way to make a patch better) leaves the optimal residence time unchanged (Charnov and Parker 1995; Ranta et al. 1995; Livoreil and Giraldeau 1997). Even one of the most basic predictions attributed to the MVT, that increasing travel time should increase optimal residence time, may not hold in all generality (Stephens and Krebs 1986). This is a concern, since such predictions are often used as a basis to evaluate the theory (e.g. Nonacs 2001; Wajnberg et al. 2006; Hayden et al. 2011).

In this article, we propose to derive general analytical predictions on the impact of varying habitat attributes under the MVT. By using sensitivity analysis on the implicit definition of optimal strategies, we do not have to specify specific functional forms and thus retain the original generality of the Theorem. This will allow us to refine and clarify existing predictions, and to generate novel predictions. In particular, our approach can deal with the arguably more general case of heterogeneous habitats, allowing for a systematic analysis of the consequences of habitat heterogeneity. We will use our results to reanalyze the main predictions attributed to the MVT, in particular the effect of varying travel time, the consequences of improving quality, the invariance of the optimal strategies upon vertical and horizontal scalings, and the relative time individuals should spend on patches of different qualities.

## The Marginal Value Theorem

Consider an individual foraging over many discrete patches that are encountered sequentially, with characteristics drawn randomly from a stationary distribution. Let there be $s$ different types of patches, each with relative frequency $p_{i}$. Let function $F_{i}(t)$ be the cumulated gain of an individual that exploits a patch of type $i$ for $t$ time units. Functions $F_{i}$ should represent net expected gains, discounting costs (Stephens and Krebs 1986; Brown 1988). They must be positive, increasing, and concave for at least some $t$ in order to yield a fitness maximum (Charnov 1976). Let $T_{i}$ be the travel time it takes to find and move to a patch of type $i$, allowing the possibility for some patches to be more accessible than others.

In a homogeneous habitat, $F_{i}=F$ and $T_{i}=T$ for all $i$. The MVT states that an individual should leave a patch after $t^{*}$ time units, as defined by

1 $$\begin{aligned} \left. \frac{dF(t)}{dt}\right| _{t = t^{*}}=\frac{F(t^{*})}{T+t^{*}}. \end{aligned}$$

**Fig. 1:**
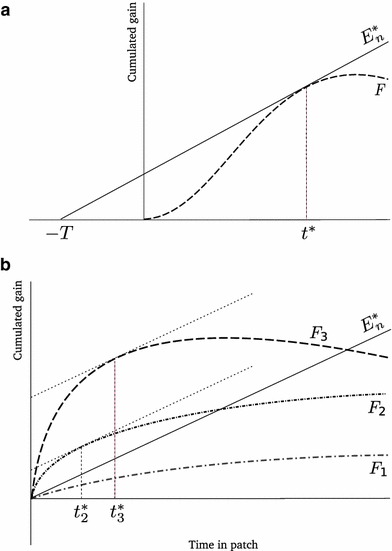
Graphical interpretation of the MVT. **a** In homogeneous habitats, (4) can be solved for $t^{*}$ by constructing the line tangential to $F$ as shown. The resulting line has slope $E_{n}^{*}$, the realized fitness. **b** In heterogeneous habitats the graphical construct does not work to solve (5). If $E_{n}^{*}$ is known, the optimal residence times on each patch-type can be determined by constructing lines tangential to the gain functions with slope $E_{n}^{*}$. Here there are three patch-types and the first is not effectively exploited, i.e. $\Omega =\left\{ 2,3\right\}$. In this case patch-type 3 has higher quality than patch-type 2, and $t_{3}^{*}>t_{2}^{*}$

Both sides are then equal to $E_{n}^{*}$, the long term average rate of gain in the habitat, which effectively represents fitness and is maximized at the optimal residence time $t^{*}$ (Charnov 1976). Equation (1) has a well-known graphical solution (Fig. 1).

In heterogeneous habitats, the MVT states that at one or more patch-types (whose indices make up the set $\Omega$) should be exploited, while others should be left as soon as entered. We denote the average value of quantity $y$ over the habitat as

2 $$\begin{aligned} \left\langle y_{j}\right\rangle =\sum _{j=1}^{s}p_{j}y_{j}. \end{aligned}$$

In some contexts the average should be over the exploited patches only. This will be made clear with a $\Omega$ subscript: $\left\langle y_{j}\right\rangle _{\Omega }=\sum _{j\in \Omega }p_{j}y_{j}/\!\sum _{j\in \Omega }p_{j}$.

The optimal residence times are then defined by

3 $$\begin{aligned} \left\{ \begin{array}{l@{\quad }l@{\quad }l} \left. \frac{dF_{i}(t_{i})}{dt_{i}}\right| _{t_{i}=t_{i}^{*}}=\frac{\left\langle F_{j}(t_{j}^{*})\right\rangle }{\left\langle T_{j}+t_{j}^{*}\right\rangle } &{} &{} i\in \Omega \\ t_{i}^{*}=0 &{} &{} i\notin \Omega \end{array} \right. \end{aligned}$$

For exploited patch-types, both sides of (3) are again equal to $E_{n}^{*}$, the fitness of an optimal individual in this habitat. Set $\Omega$ is determined as the set that satisfies (3) while resulting in the highest value of $E_{n}^{*}$ (Charnov 1976; Stephens and Krebs 1986). There is no graphical solution in this case, even though if $E_{n}^{*}$ has been determined, one can still deduce the optimal residence times on each patch-type (Fig. 1).

In order to determine the consequences of changing habitat characteristics, we introduce an indicator variable $x$ that represents some relevant attribute of patches. Different attributes (e.g. patch size, nutritional value...) can be relevant depending on context (Charnov and Parker 1995; Rita et al. 1997). Attributes of interest would typically impact the shape of the gain function (McNair 1982) and/or travel time (Lundberg and Danell 1990; Charnov and Parker 1995). In this context, the homogeneous MVT Eq. (1) can be expressed as

4 $$\begin{aligned} \frac{\partial F(x,t)}{\partial t}=\frac{F(x,t)}{T(x)+t}, \end{aligned}$$

evaluated at the MVT optimum $\left( x_{0},t^{*}(x_{0})\right)$.

The heterogeneous Eq. (3) becomes

5 $$\begin{aligned} \left\{ \begin{array}{l@{\quad }l@{\quad }l} \frac{\partial F_{i}(x_{i},t_{i})}{\partial t_{i}}=\frac{\left\langle F_{j}(x_{j},t_{j})\right\rangle }{\left\langle T_{j}(x_{j})+t_{j}\right\rangle } &{} &{} i\in \Omega \\ t_{i}^{*}=0 &{} &{} i\notin \Omega \end{array} \right. \end{aligned}$$

evaluated at $\mathbf {x_{0}}=\left( x_{01}, \ldots , x_{0j}, \ldots ,x_{0s}\right)$ and $\mathbf {t^{*}}(\mathbf {x_{0}})=\Big (t_{1}^{*}(\mathbf {x_{0}}), \ldots , t_{j}^{*}(\mathbf {x_{0}}), \ldots ,t_{s}^{*}(\mathbf {x_{0}})\Big )$.

For generality, all functions $F$ and $T$ in (4) and (5) will be assumed to be sufficiently smooth in their arguments. We will also assume that there exists only one MVT optimum in a given habitat. We will study the consequences of slightly varying the $x_{0}$ values on the MVT optimum defined from (4)/(5). In order to reduce clutter, we will simply recall that expressions are evaluated at the MVT optimum by noting $t_{i}^{*}$ in lieu of $t_{i}$.

## Realized fitness, or what is quality under the MVT

The notion of quality is seldom made precise in the context of the MVT. Quality is sometimes equated with accessibility or connectivity (Thompson and Fedak 2001; Belisle 2005; Nolet and Klaassen 2009), so that higher quality implies shorter travel time. On the other hand, better patches are often considered to be those with more resources, and hence higher gains. However, there is no unique way to ’improve’ a gain function. In this article, we remark that for an optimal forager, an objective measure of habitat quality is the realized fitness $E_{n}^{*}$, i.e. the long-term rate of gain it extracts from its habitat. Hence, we consider than any alteration of the habitat corresponds to improving quality if it increases the realized fitness $E_{n}^{*}$. In particular, regarding the choice of $x_{i}$:

### **Definition 1**

In a given habitat, a patch-attribute $x_{i}$ is called a metric of quality if and only if $\partial E_{n}^{*}/\partial x_{i}>0$.

We now proceed to compute $\partial E_{n}^{*}/\partial x_{i}$ from (5), in order to clarify which sorts of patch alterations result in improved quality.

### **Proposition 1**

A patch attribute $x_{i}$ is a metric of quality (Definition 1) if and only if

6 $$\begin{aligned} \frac{\partial \ln \left\langle F_{j}(x_{j},t_{j}^{*})\right\rangle }{\partial x_{i}}-\frac{\partial \ln \left\langle T_{j}(x_{j})\right\rangle }{\partial x_{i}}\frac{\left\langle T_{j}(x_{j})\right\rangle }{\left\langle T_{j}(x_{j})+t_{j}^{*}\right\rangle }>0. \end{aligned}$$

### *Proof*

From the expression of the realized fitness in (5), $E_{n}^{*}=\langle F_{j}(x_{j},t_{j}^{*})\rangle /\langle T_{j}(x_{j})+t_{j}^{*}\rangle$, we see that it is affected by $x_{i}$ directly through the effect on $F_{j}$ and $T_{j}$, and indirectly through the effect on $\mathbf {t^{*}}$. We can thus express the variation of $E_{n}^{*}$ as

$$\begin{aligned} \frac{\partial E_{n}^{*}}{\partial x_{i}}=\frac{\partial }{\partial x_{i}}\frac{\left\langle F_{j}(x_{j},t_{j}^{*})\right\rangle }{\left\langle T_{j}(x_{j})+t_{j}^{*}\right\rangle }+\sum _{l=1}^{s}\left( \frac{\partial }{\partial t_{l}}\frac{\left\langle F_{j}(x_{j},t_{j}^{*})\right\rangle }{\left\langle T_{j}(x_{j})+t_{j}^{*}\right\rangle }\right) \frac{\partial t_{l}^{*}}{\partial x_{i}}. \end{aligned}$$

Each derivative with respect to $t_{l}$ (second term on the r.h.s.) is zero, as the long term average rate of gain $E_{n}^{*}$ is maximized at $\mathbf {t^{*}}(\mathbf {x_{0}})$ under the MVT. Hence, all terms involving variations of the optimal residence times vanish. Expanding the remaining terms yields:

$$\begin{aligned} \frac{\partial E_{n}^{*}}{\partial x_{i}}=\frac{1}{\left\langle T_{j}(x_{j})+t_{j}^{*}\right\rangle ^{2}}\left( p_{i}\left\langle T_{j}(x_{j})+t_{j}^{*}\right\rangle \frac{\partial F_{i}(x_{i},t_{i}^{*})}{\partial x_{i}}-p_{i}\left\langle F_{j}(x_{j},t_{j}^{*})\right\rangle \frac{dT_{i}(x_{i})}{dx_{i}}\right) . \end{aligned}$$

Remembering the expression of $E_{n}^{*}$ (from (5)), this simplifies as:

7 $$\begin{aligned} \frac{\partial E_{n}^{*}}{\partial x_{i}}=\frac{p_{i}}{\left\langle T_{j}(x_{j})+t_{j}^{*}\right\rangle }\left( \frac{\partial F_{i}(x_{i},t_{i}^{*})}{\partial x_{i}}-E_{n}^{*}\frac{dT_{i}(x_{i})}{dx_{i}}\right) . \end{aligned}$$

We divide both sides by $E_{n}^{*}$, and remark that

$$\begin{aligned} p_{i}\frac{\partial F_{i}(x_{i},t_{i})}{\partial x_{i}}=\frac{\partial }{\partial x_{i}}\left\langle F_{j}(x_{j},t_{j})\right\rangle \;\mathrm{ and }\; p_{i}\frac{dT_{i}(x_{i})}{dx_{i}}=\frac{\partial }{\partial x_{i}}\left\langle T_{j}(x_{j})\right\rangle , \end{aligned}$$

which, upon taking the logarithms, yields the relative variation of $E_{n}^{*}$ in terms of the relative variation of average quantities:

8 $$\begin{aligned} \frac{\partial \ln E_{n}^{*}}{\partial x_{i}}=\frac{\partial \ln \left\langle F_{j}(x_{j},t_{j}^{*})\right\rangle }{\partial x_{i}}-\frac{\partial \ln \left\langle T_{j}(x_{j})\right\rangle }{\partial x_{i}}\frac{\left\langle T_{j}(x_{j})\right\rangle }{\left\langle T_{j}(x_{j})+t_{j}^{*}\right\rangle }. \end{aligned}$$

Requiring this to be positive yields Proposition 1. $\square$

Equation (8) states that the variation of realized fitness only depends on the relative variations of average absolute gains (first term) and of average travel time (second term). Changing the time-derivative of the fitness function (i.e. the instantaneous rate of gain) has no direct impact on fitness; only absolute gains matter. It is therefore unduely restrictive to assume better patches have steeper slopes with respect to time, as in some earlier analyses (e.g. McNair 1982). The slope of the fitness functions might vary arbitrarily with quality, as it will prove important throughout this article.

We also remark that the relative variation of average travel time is weighted by $\langle T_{j}(x_{j})\rangle /\langle T_{j}(x_{j})+t_{j}^{*}\rangle$ (8). Since this represents the proportion of time an individual spends traveling between patches, it is necessarily smaller than one. Hence, a relative increase in average travel time does not compensate for a similar relative increase in the average gains. In other words, travel time has comparatively less impact than the gain function.

## Optimal residence times

### Homogeneous habitats

We now show that, in a homogeneous habitat, the effect of varying a patch-attribute $x$ depends on how this changes the time-derivative of $F$, its height, and travel time. We have the following theorem:

#### **Theorem 1**

Increasing $x$ increases $t^{*}$if and only if

9 $$\begin{aligned} \frac{\partial }{\partial x}\ln \frac{\partial F(x,t^{*})}{\partial t}-\frac{\partial \ln F(x,t^{*})}{\partial x}+\frac{d\ln T(x)}{dx}\frac{T(x)}{T(x)+t^{*}}>0 \end{aligned}$$

#### *Proof*

Since the MVT holds irrespective of habitat quality, (4) remains true if both sides are differentiated with respect to $x$, which yields:

10 $$\begin{aligned} \frac{\partial ^{2}F(x,t^{*})}{\partial x\partial t}+\frac{\partial ^{2}F(x,t^{*})}{\partial t^{2}}\frac{dt^{*}}{dx}=\frac{\partial E_{n}^{*}}{\partial x} \end{aligned}$$

Isolating the derivative of interest:

$$\begin{aligned} \frac{dt^{*}}{dx}=-\left( \frac{\partial ^{2}F(x,t^{*})}{\partial x\partial t}-\frac{\partial E_{n}^{*}}{\partial x}\right) \left( \frac{\partial ^{2}F(x,t^{*})}{\partial t^{2}}\right) ^{-1} \end{aligned}$$

The two terms in the parenthesis can be turned into relative variations by dividing them by $E_{n}^{*}=\partial F(x,t^{*})/\partial t$:

11 $$\begin{aligned} \frac{dt^{*}}{dx}=-E_{n}^{*}\left( \frac{\partial }{\partial x}\ln \frac{\partial F(x,t^{*})}{\partial t}-\frac{\partial \ln E_{n}^{*}}{\partial x}\right) \left( \frac{\partial ^{2}F(x,t^{*})}{\partial t^{2}}\right) ^{-1} \end{aligned}$$

Since function $F$ is concave at a MVT optimum, $\partial ^{2}F(x,t^{*})/\partial t^{2}<0$ and the sign of variation of $t^{*}$ is that of the first parenthesis. Replacing $\partial \ln E_{n}^{*}/\partial x$ with its value from (8) (evaluated in the homogeneous case) concludes the proof. $\square$

As was the case for realized fitness (8), travel time has relatively less impact on optimal residence time than the two attributes of the gain function. This follows directly from (9) in which the relative variation of travel time is weighted down by $T(x)/(T(x)+t^{*})<1$.

One consequence of Theorem 1 is that an increase in quality may increase the optimal residence time only if it increases sufficiently the slope of the gain function. This follows directly from (11), since $\partial \ln E_{n}^{*}/\partial x$ is positive for any metric of quality $x$ (Definition 1).

### Heterogeneous habitats

In heterogeneous habitats, the optimal residence time on patch-type $i$ is affected not only by the attribute of patch-type $i$, but also by the attributes of all other patches. We have the following result:

#### **Theorem 2**

In a heterogeneous habitat, for any $i\in \{1,\ldots ,s\}$ and $k\in \Omega$, increasing $x_{i}$ increases $t_{k}^{*}$ if and only if

12 $$\begin{aligned} \frac{\partial }{\partial x_{i}}\ln \frac{\partial F_{k}(x_{k},t_{k}^{*})}{\partial t_{k}}-\frac{\partial \ln \left\langle F_{j}(x_{j},t_{j}^{*})\right\rangle }{\partial x_{i}}+\frac{\partial \ln \left\langle T_{j}(x_{j})\right\rangle }{\partial x_{i}}\frac{\left\langle T_{j}(x_{j})\right\rangle }{\left\langle T_{j}(x_{j})+t_{j}^{*}\right\rangle }>0\qquad \qquad \end{aligned}$$

#### *Proof*

For any patch-type $m$ not in $\Omega$ , $t_{m}^{*}=0$ and, generically, it does not vary with $\mathbf {x}$, i.e. $\partial t_{m}^{*}/\partial x_{i}=0$ for all $i$. Let us consider the variation of $t_{k}^{*}$, $k\in \Omega$, with respect to the attribute of some patch-type $i$. We use Eq. (5), replacing $i$ with $k$, and differentiate both sides with respect to $x_{i}$, to get

$$\begin{aligned} \frac{\partial ^{2}F_{k}(x_{k},t_{k}^{*})}{\partial x_{i}\partial t_{k}}+\frac{\partial ^{2}F_{k}(x_{k},t_{k}^{*})}{\partial t_{k}^{2}}\frac{\partial t_{k}^{*}}{\partial x_{i}}=\frac{\partial E_{n}^{*}}{\partial x_{i}} \end{aligned}$$

The same rearrangements as above yield:

13 $$\begin{aligned} \frac{\partial t_{k}^{*}}{\partial x_{i}}=-E_{n}^{*}\left( \frac{\partial }{\partial x_{i}}\ln \frac{\partial F_{k}(x_{k},t_{k}^{*})}{\partial t_{k}}-\frac{\partial \ln E_{n}^{*}}{\partial x_{i}}\right) \left( \frac{\partial ^{2}F_{k}(x_{k},t_{k}^{*})}{\partial t_{k}^{2}}\right) ^{-1}\qquad \end{aligned}$$

Replacing $\partial \ln E_{n}^{*}/\partial x_{i}$ with its value from Eq. (8), we get the condition for $t_{k}^{*}$ to increase with $x_{i}$ as expressed in Theorem 2. $\square$

#### **Corollary 1**

For any $i\in \{1,\ldots ,s\}$ and $k\in \Omega$, $k\ne i$, if $x_{i}$ is a metric of quality increasing $x_{i}$ decreases $t_{k}^{*}$.

#### *Proof*

We remark that, in the absence of further assumptions, $\partial ^{2}F_{k}(x_{k},t_{k}^{*})/\partial x_{i}\partial t_{k}=0$ for any $k\ne i$. Hence, the proposition is a direct consequence of Theorem 2, as $\partial E_{n}^{*}/\partial x_{i}>0$ if $x_{i}$ is a metric of quality. $\square$

Equation (13) includes the homogeneous case studied in the previous section as a special case. It is thus insightful to compare the value of $\partial t_{i}^{*}/\partial x_{i}$, for one patch-type $i$, depending on whether the habitat is homogeneous or heterogeneous. For this, we consider as known all quantities observable at the patch level, i.e. $t_{i}^{*}$ (and thus $E_{n}^{*}$), $F_{i}(x_{0i},t)$ and, if relevant, $T_{i}(x_{0i})$, but let the habitat context ($p_{i}$ and the attributes of other patches) unspecified. When considering attributes of quality, this yields the following proposition:

#### **Proposition 2**

In a habitat of quality $E_{n}^{*}$, the variation of $t_{i}^{*}$ with a quality metric $x_{i}$ is always greater if the habitat is heterogeneous rather than homogeneous. In heterogeneous habitats, $\partial t_{i}^{*}/\partial x_{i}$ is lower the smaller $p_{i}$, or the larger $\langle F_{j}(x_{j},t_{j}^{*})\rangle$ relative to $F_{i}(x_{i},t_{i}^{*})$.

#### *Proof*

Consider a habitat of quality $E_{n}^{*}$ and a focal patch-type $i$ with attributes $F_{i}$($x_{0i}$,t) and $T_{i}$($x_{0i}$), so that $t_{i}^{*}$ is fixed. If the habitat is homogeneous, $\partial t_{i}^{*}/\partial x_{i}$ is given by (11) applied to patch-type $i$, whereas if it is heterogeneous, $\partial t_{i}^{*}/\partial x_{i}$ is given by (13). The two equations are almost identical, differing only in the relative variation of $E_{n}^{*}$. In the heterogeneous case, the latter is:

$$\begin{aligned} -\frac{\partial \ln E_{n}^{*}}{\partial x_{i}}=-\frac{p_{i}}{\left\langle F_{j}(x_{j},t_{j}^{*})\right\rangle }\frac{\partial F_{i}(x_{i},t_{i}^{*})}{\partial x_{i}}+\frac{p_{i}}{\left\langle T_{j}(x_{j})+t_{j}^{*}\right\rangle }\frac{dT_{i}(x_{i})}{dx_{i}} \end{aligned}$$

which can be rewritten as

14 $$\begin{aligned} -p_{i}\left( \frac{F_{i}(x_{i},t_{i}^{*})}{\left\langle F_{j}(x_{j},t_{j}^{*})\right\rangle }\frac{1}{F_{i}(x_{i},t_{i}^{*})}\frac{\partial F_{i}(x_{i},t_{i}^{*})}{\partial x_{i}}-\frac{T_{i}(x_{i})+t_{i}^{*}}{\left\langle T_{j}(x_{j})+t_{j}^{*}\right\rangle }\frac{1}{T_{i}(x_{i})+t_{i}^{*}}\frac{dT_{i}(x_{i})}{dx_{i}}\right) \nonumber \\ \end{aligned}$$

For $E_{n}^{*}$ to be the same in the homogeneous and heterogeneous cases, we must have

$$\begin{aligned} \frac{F_{i}(x_{i},t_{i}^{*})}{T_{i}(x_{i})+t_{i}^{*}}=\frac{\left\langle F_{j}(x_{j},t_{j}^{*})\right\rangle }{\left\langle T_{j}(x_{j})+t_{j}^{*}\right\rangle }\Leftrightarrow \frac{F_{i}(x_{i},t_{i}^{*})}{\left\langle F_{j}(x_{j},t_{j}^{*})\right\rangle }=\frac{T_{i}(x_{i})+t_{i}^{*}}{\left\langle T_{j}(x_{j})+t_{j}^{*}\right\rangle } \end{aligned}$$

From this, Eq. (14) simplifies as

$$\begin{aligned} -\frac{\partial \ln E_{n}^{*}}{\partial x_{i}}=-p_{i}\frac{F_{i}(x_{i},t_{i}^{*})}{\left\langle F_{j}(x_{j},t_{j}^{*})\right\rangle }\left( \frac{1}{F_{i}(x_{i},t_{i}^{*})}\frac{\partial F_{i}(x_{i},t_{i}^{*})}{\partial x_{i}}-\frac{1}{T_{i}(x_{i})+t_{i}^{*}}\frac{dT_{i}(x_{i})}{dx_{i}}\right) \end{aligned}$$

This is the same as in a homogeneous habitat (Eq. 11), multiplied by $p_{i}F_{i}(x_{i},t_{i}^{*})/\langle F_{j}(x_{j},t_{j}^{*})\rangle \le 1$. If $x_{i}$ is a quality-metric, $-\partial \ln E_{n}^{*}/\partial x_{i}<0$ by definition, so that $\partial t_{i}^{*}/\partial x_{i}$ from (12) is no smaller than from (11), with equality in the homogeneous case. The difference decreases in proportion of $p_{i}$, and in inverse proportion of $\langle F_{j}(x_{j},t_{j}^{*})\rangle$, concluding the proof. $\square$

Intuitively, Proposition 2 means that the habitat acts as a diluting factor, buffering the impact of patch attributes on the overall habitat quality $E_{n}^{*}$. The greater the contribution of patch-type $i$ to the overall quality, i.e. the higher $p_{i}$ and $F_{i}(x_{i},t_{i}^{*})$, the greater the variation of $E_{n}^{*}$ with $x_{i}$, which feedbacks negatively on $t_{i}^{*}$. Homogeneous habitats represent an ideal case where the retroaction of $E_{n}^{*}$ on $t_{i}^{*}$ has full intensity, maximizing the chances of having a negative $\partial t_{i}^{*}/\partial x_{i}$.

### Average residence time

Comparing equations (11) and (13) helped evaluate the consequences of habitat heterogeneity from the perspective of a focal patch-type. From a whole-habitat perspective, a more meaningful comparison is between the behavior of $t^{*}$ in the homogeneous case and that of $\langle t_{j}^{*}\rangle$ in the heterogeneous case. Indeed, $t^{*}$ and $\langle t_{j}^{*}\rangle$ both capture the global rate of movement throughout the habitat. One question of interest is whether heterogeneous habitats behave on average just like an average homogeneous habitat, so that one might just plug average quantities into Eq. (9), or whether heterogeneity changes things qualitatively.

#### **Theorem 3**

In a heterogeneous habitat,  for any $i\in \{1,\ldots ,s\}$, increasing $x_{i}$ increases $\langle t_{j}^{*}\rangle$ if and only if

$$\begin{aligned} \left( \frac{\partial ^{2}F_{i}(x_{i},t_{i}^{*})}{\partial t_{i}^{2}}\right) ^{-1}\frac{\partial }{\partial x_{i}}\ln \left\langle \frac{\partial F_{j}(x_{j},t_{j}^{*})}{\partial t_{j}}\right\rangle _{\Omega }-\left\langle \left( \frac{\partial ^{2}F_{j}(x_{j},t_{j}^{*})}{\partial t_{j}^{2}}\right) ^{-1}\right\rangle _{\Omega }\frac{\partial \ln E_{n}^{*}}{\partial x_{i}}>0 \end{aligned}$$

#### *Proof*

We compute the variation of average residence time with $x_{i}$:

$$\begin{aligned} \frac{\partial \left\langle t_{j}^{*}\right\rangle }{\partial x_{i}}=\left\langle \frac{\partial t_{j}^{*}}{\partial x_{i}}\right\rangle =\sum _{k\notin \Omega }p_{k}\frac{\partial t_{k}^{*}}{\partial x_{i}}+\sum _{k\in \Omega }p_{k}\frac{\partial t_{k}^{*}}{\partial x_{i}} \end{aligned}$$

For any patch $k$ not in $\Omega$ , $\partial t_{k}^{*}/\partial x_{i}=0,$ and using (13) for the others, we get

$$\begin{aligned} \frac{\partial \left\langle t_{j}^{*}\right\rangle }{\partial x_{i}}=\sum _{k\in \Omega }-p_{k}E_{n}^{*}\left( \frac{\partial }{\partial x_{i}}\ln \frac{\partial F_{k}(x_{k},t_{k}^{*})}{\partial t_{k}}-\frac{\partial \ln E_{n}^{*}}{\partial x_{i}}\right) \left( \frac{\partial ^{2}F_{k}(x_{k},t_{k}^{*})}{\partial t_{k}^{2}}\right) ^{-1}, \end{aligned}$$

which leads to

15 $$\begin{aligned} \frac{\partial \left\langle t_{j}^{*}\right\rangle }{\partial x_{i}}&= E_{n}^{*}\left( p_{i}\left( -\frac{\partial }{\partial x_{i}}\ln \frac{\partial F_{i}(x_{i},t_{i}^{*})}{\partial t_{i}}\right) \left( \frac{\partial ^{2}F_{i}(x_{i},t_{i}^{*})}{\partial t_{i}^{2}}\right) ^{-1}\right. \nonumber \\&\left. +\,\frac{\partial \ln E_{n}^{*}}{\partial x_{i}}\sum _{k\in \Omega }p_{k}\left( \frac{\partial ^{2}F_{k}(x_{k},t_{k}^{*})}{\partial t_{k}^{2}}\right) ^{-1}\right) . \end{aligned}$$

Here we remark that, from (2) and the definition of $\left\langle y_{j}\right\rangle _{\Omega }$,

$$\begin{aligned} p_{i}\frac{\partial }{\partial x_{i}}\frac{\partial F_{i}(x_{i},t_{i}^{*})}{\partial t_{i}}=\frac{\partial }{\partial x_{i}}\left\langle \frac{\partial F_{j}(x_{j},t_{j}^{*})}{\partial t_{j}}\right\rangle =\left( \sum _{k\in \Omega }p_{k}\right) \frac{\partial }{\partial x_{i}}\left\langle \frac{\partial F_{j}(x_{j},t_{j}^{*})}{\partial t_{j}}\right\rangle _{\Omega } \end{aligned}$$

and, since, for $i\in \Omega$, $\partial F_{i}(x_{i},t_{i}^{*})/\partial t_{i}=E_{n}^{*}$,

$$\begin{aligned} \left\langle \frac{\partial F_{j}(x_{j},t_{j}^{*})}{\partial t_{j}}\right\rangle _{\Omega }=E_{n}^{*}. \end{aligned}$$

Thus, we have

$$\begin{aligned} p_{i}\frac{\partial }{\partial x_{i}}\ln \frac{\partial F_{i}(x_{i},t_{i}^{*})}{\partial t_{i}}=\left( \sum _{k\in \Omega }p_{k}\right) \frac{\partial }{\partial x_{i}}\ln \left\langle \frac{\partial F_{j}(x_{j},t_{j}^{*})}{\partial t_{j}}\right\rangle _{\Omega }. \end{aligned}$$

Also, we can write

$$\begin{aligned} \sum _{k\in \Omega }p_{k}\left( \frac{\partial ^{2}F_{k}(x_{k},t_{k}^{*})}{\partial t_{k}^{2}}\right) ^{-1}=\left( \sum _{k\in \Omega }p_{k}\right) \left\langle \left( \frac{\partial ^{2}F_{j}(x_{j},t_{j}^{*})}{\partial t_{j}^{2}}\right) ^{-1}\right\rangle _{\Omega }. \end{aligned}$$

Using these in Eq. (15) yields:

16 $$\begin{aligned} \frac{\partial \left\langle t_{j}^{*}\right\rangle }{\partial x_{i}}&= -E_{n}^{*}\left( \sum _{k\in \Omega }p_{k}\right) \left[ \left( \frac{\partial ^{2}F_{i}(x_{i},t_{i}^{*})}{\partial t_{i}^{2}}\right) ^{-1}\frac{\partial }{\partial x_{i}}\ln \left\langle \frac{\partial F_{j}(x_{j},t_{j}^{*})}{\partial t_{j}}\right\rangle _{\Omega }\right. \nonumber \\&\left. -\left\langle \left( \frac{\partial ^{2}F_{j}(x_{j},t_{j}^{*})}{\partial t_{j}^{2}}\right) ^{-1}\right\rangle _{\Omega }\frac{\partial \ln E_{n}^{*}}{\partial x_{i}}\right] . \end{aligned}$$

Requiring this to be positive establishes the theorem. $\square$

Unlike in a homogeneous habitat, one cannot in general obtain a criterion for the sign of $\partial \langle t_{j}^{*}\rangle /\partial x_{i}$ that does not require estimating second time-derivatives. Indeed, the first term in square brackets in (16) is divided by $\partial ^{2}F_{i}(x_{i},t_{i}^{*})/\partial t_{i}^{2}$ while the second is divided by

17 $$\begin{aligned} \left\langle \left( \frac{\partial ^{2}F_{j}(x_{j},t_{j}^{*})}{\partial t_{j}^{2}}\right) ^{-1}\right\rangle _{\Omega }^{-1}=-H_{\Omega }\left( \frac{-\partial ^{2}F_{j}(x_{j},t_{j}^{*})}{\partial t_{j}^{2}}\right) \end{aligned}$$

where $H$ is the harmonic (rather than arithmetic) mean over exploited patches. Since, at the optimal residence times, all time-derivatives in $\Omega$ are equal to $E_{n}^{*}$, second time-derivatives are effectively proportional to the curvatures w.r.t. time. The harmonic mean (17) is thus the appropriate way to average curvatures in the context of the MVT.

Curvatures can be disregarded if all are identical at the MVT optimum, or if the manipulated patch-type $i$ has exactly average curvature. These may seem very contrived situations, but we will encounter an occurrence of the former in Sect. 5.3, which is also found in a broader context of biological relevance (Calcagno et al. *in prep*.). In these circumstances, minus the harmonic mean is equal to the second time-derivative of $F_{i}$ so that both can be factored out in (16), yielding the condition for $\partial \langle t_{j}^{*}\rangle /\partial x_{i}$ to be positive as:

18 $$\begin{aligned} \frac{\partial }{\partial x_{i}}\ln \left\langle \frac{\partial F_{j}(x_{j},t_{j}^{*})}{\partial t_{j}}\right\rangle _{\Omega }-\frac{\partial \ln E_{n}^{*}}{\partial x_{i}}>0. \end{aligned}$$

This is equivalent to criterion (9), stated in terms of habitat-level averages (the first only covering $\Omega$).

In general, however, a given change of average habitat characteristics might have contrasted impacts on the average optimal residence time, depending on the distribution of second-time derivatives, and on which patch-type is altered. If $x_{i}$ is a metric of quality, Theorem 3 implies that $\langle t_{j}^{*}\rangle$ might increase with $x_{i}$ only if $\partial ^{2}F_{i}(x_{i},t_{i}^{*})/\partial x_{i}\partial t_{i}$ is sufficiently positive. As the latter impacts $\langle t_{j}^{*}\rangle$ in inverse proportion of the second time derivative $\partial ^{2}F_{i}(x_{i},t_{i}^{*})/\partial t_{i}^{2}$, it follows that an increase of $\langle t_{j}^{*}\rangle$ is more likely, all else equal, when altering patch-types whose gain functions are relatively less curved.

## Applications

### Manipulating travel time

A graphical argument (corresponding to pushing $-T$ to the right in Fig. 1) is often used to predict that decreasing the travel time should shorten the optimal residence time, and thus increase movement (e.g. Danchin et al. 2008). This is possibly the simplest and most often tested prediction attributed to the MVT (Nonacs 2001; Hayden et al. 2011). However, the graphical argument works only for homogeneous habitats, and assumes that the gain functions are not affected by changes in travel time. This is not the case if there are costs associated with traveling between patches (e.g. energetic locomotory costs). These make the net gain function vary with $T$, so that the argument cannot be relied on (Stephens and Krebs 1986). We use our results to address this issue formally.

Consider the cost of moving between patches is given by an increasing function of travel time $C_{T}(T)$, while foraging costs in a patch are given by an increasing function $C_{F}(t)$. We thus have to consider the class of gain functions

19 $$\begin{aligned} F_{i}(x_{i},t)=F_{0i}(t)-C_{F}(t)-C_{T}(T_{i}(x_{i})), \end{aligned}$$

where $F_{0i}(t)$ is some function representing the gross gains in patch-type $i$.

In this context,

$$\begin{aligned} \frac{\partial \ln F_{i}(x_{i},t)}{\partial x_{i}}=-\frac{T_{i}(x_{i})}{F_{0i}(t)}\frac{dC_{T}(T)}{dT}\frac{d\ln T_{i}(x_{i})}{dx_{i}}, \end{aligned}$$

so that, from Eq. (7):

$$\begin{aligned} \frac{\partial \ln E_{n}^{*}}{\partial x_{i}}=\frac{d\ln T_{i}(x_{i})}{dx_{i}}\left( -\frac{1}{\left\langle F_{j}(x_{j},t_{j}^{*})\right\rangle }\frac{T_{i}(x_{i})}{F_{0i}(t_{i}^{*})}\frac{dC_{T}(T)}{dT}-\frac{\left\langle T_{j}(x_{j})\right\rangle }{\left\langle T_{j}(x_{j})+t_{j}^{*}\right\rangle }\right) . \end{aligned}$$

As the term in parenthesis is always negative, $E_{n}^{*}$ varies in opposite direction of travel time, and $x_{i}$ is a metric quality if and only if $T_{i}$ is decreasing in $x_{i}$.

From Eq. (19) we further have

$$\begin{aligned} \frac{\partial }{\partial x_{i}}\ln \frac{\partial F_{i}(x_{i},t)}{\partial t}=0. \end{aligned}$$

From Theorem 2, this implies that the sign of $\partial t_{k}^{*}/\partial x_{i}$, for all $i\in \{1,\ldots ,s\}$ and $k\in \Omega$, is that of $-\partial E_{n}^{*}/\partial x_{i}$. Hence optimal residence times invariably increase with travel time, proving the graphical prediction in a general setting.

### Manipulating patch frequencies

In the previous application, the time-derivatives of the gain functions were unaffected by the habitat modification; the sign of the variation of optimal residence times was thus entirely governed by the variation of $E_{n}^{*}$ (Theorem 2). We remark here that a similar situation arises when one manipulates the relative frequency of patch-types (i.e. the $p_{i}$). Whereas most applications of the MVT have investigated the consequences of changing patch-attributes (Stephens and Krebs 1986), changing the abundance of different patch-types constitutes a general alternative way to alter a habitat.

For clarity, let us omit the dependence of $F_{i}$ and $T_{i}$ on $x_{i}$. If we consider a change in $p_{i}$, at least one other $p_{k}$ must also change in order to maintain $\sum _{j}p_{j}=1$. When differentiating Eq. (3) with respect to $p_{i}$, we thus have to take the total derivatives with respect to $p_{i}$. We get, for all $i\in \{1,\ldots ,s\}$ and $k\in \Omega$,

20 $$\begin{aligned} \frac{d^{2}F_{k}(t_{k}^{*})}{dt_{k}^{2}}\frac{dt_{k}^{*}}{dp_{i}}=\frac{dE_{n}^{*}}{dp_{i}}. \end{aligned}$$

Since $d^{2}F_{k}(t_{k}^{*})/dt_{k}^{2}<0$ at any MVT optimum, this immediately shows that $dt_{k}^{*}/dp_{i}$ has the sign of $-dE_{n}^{*}/dp_{i}$. Hence, improving habitat quality by manipulating relative patch frequencies decreases all patch residence times, i.e. increases the movement rate. This is another illustration that, if the time-derivatives of the gain functions are left unchanged, the optimal residence times on exploited patches invariably decrease with $E_{n}^{*}$.

### On the scaling invariance of optimal strategies

We now consider two forms of scaling invariance of the optimal strategies that have been attributed to the MVT based on particular functions, the first corresponding to scaling the gain function vertically (i.e. scaling the gains), the second corresponding to scaling time (including travel time). Two particular gain functions are often used to implement these scenarios, namely the negative exponential function

21 $$\begin{aligned} \mu (1-\exp (-\lambda t)), \end{aligned}$$

and the Michaelis–Menten function

22 $$\begin{aligned} v_{m}t/(k+t). \end{aligned}$$

#### Scaling the gains

A generic way to model an increase in the quality of a patch is to multiply its gain function by some constant greater than one, effectively “stretching” it vertically. This can represent a change in the per-capita value of resource items (such as the sugar concentration in nectar or honeydew; Bonser et al. 1998), a change in their sheer number (Parker and Stuart 1976; Wajnberg et al. 2006), or the increased harvesting rate when more social foragers work together on a patch (Ranta et al. 1995; Livoreil and Giraldeau 1997). This has traditionally been modelled as increasing parameters $\mu$ and $v_{m}$ in functions (21) and (22).

**Fig. 2:**
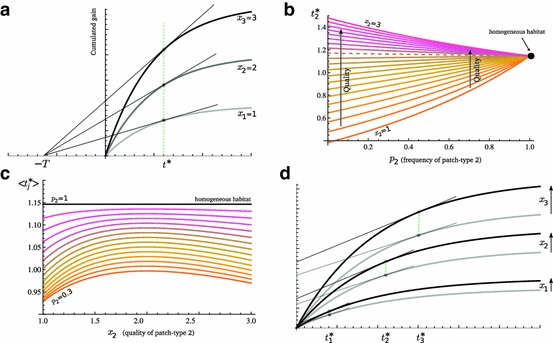
Scaling the gains and invariance. Function (21) is used as an illustration of class (23). **a** For homogeneous habitats, holding travel time fixed at $T$, $t^{*}$ is invariant to quality (*green vertical line*). **b** $t_{i}^{*}$ always increases with $x_{i}$ (*vertical arrows*), except in the homogeneous case (*dot on the far right*). In this example $x_{2}$ and $p_{2}$ were varied in a habitat with two other patch-types, having qualities $x_{1}=1$ and $x_{3}=3$, and relative frequencies $p_{1}=p_{3}=(1-p_{2})/2$. Remark that increasing $p_{2}$ increases $t_{2}^{*}$ if it decreases $E_{n}^{*}$ (low $x_{2}$ values, *below dotted curve*) and decreases $t_{2}^{*}$ otherwise (high $x_{2}$ values, *above dotted curve*). **c** Except in the homogeneous case (*black line*), the average residence time varies with $x_{2}$. It increases for low $x_{2}$ values and decreases for high $x_{2}$ values. **d** In a heterogeneous habitat, if all gain functions are scaled by the same factor, optimal residence times do not vary. In this example, all $x$ values where multiplied by $\frac{5}{4}$ (from *gray to black*). *Other parameters*: $T=1$, $\lambda =1$ (color figure online)

From the latter functions, it has been found that $t^{*}$ does not vary with $x$ in homogeneous habitats, if travel time is kept constant (Stephens and Dunbar 1993; Charnov and Parker 1995; Ranta et al. 1995). A graphical illustration is given in Fig. 2a. We will here establish this result in a more general setting, and show that this invariance is non-generic when one considers habitat heterogeneity.

We will consider the following class of gain functions:

23 $$\begin{aligned} F_{i}(x_{i},t)=x_{i}G_{i}(t), \end{aligned}$$

with $x_{i}>0$ and some arbitrary functions $G_{i}$, and constant travel times, i.e. $dT_{i}(x_{i})/dx_{i}=0$. Class (23) includes both (21) and (22), with $x$ taken to be $\mu$ and $v_{m}$, respectively.

We first remark that $F_{i}(x_{i},t_{i}^{*})$ must be positive at a MVT optimum, so that $\partial F_{i}(x_{i},t_{i}^{*})/\partial x_{i}=G_{i}(t_{i}^{*})>0$ for any feasible $t_{i}^{*}$. Hence, from Proposition 1, $x_{i}$ is indeed a metric of quality. Equation (23) also implies

$$\begin{aligned} \frac{\partial F_{i}(x_{i},t)}{\partial x_{i}}=\frac{\partial x_{i}G_{i}(t)}{\partial x_{i}}=G_{i}(t)\quad \mathrm{ and }\quad \frac{\partial ^{2}F_{i}(x_{i},t)}{\partial x_{i}\partial t}=\frac{dG_{i}(t)}{dt}. \end{aligned}$$

From Theorem 2, we thus have the condition for $t_{i}^{*}$ to increase with $x_{i}$, $i\in \Omega$, as

$$\begin{aligned} \frac{1}{E_{n}^{*}}\frac{dG_{i}(t_{i}^{*})}{dt}-p_{i}\frac{G_{i}(t_{i}^{*})}{\left\langle x_{j}G_{j}(t_{j}^{*})\right\rangle }>0. \end{aligned}$$

Since $E_{n}^{*}=x_{i}dG_{i}(t_{i}^{*})/dt$, this simplifies as

24 $$\begin{aligned} \frac{1}{x_{i}}-\frac{p_{i}G_{i}(t_{i}^{*})}{\left\langle x_{j}G_{j}(t_{j}^{*})\right\rangle }>0\Leftrightarrow p_{i}x_{i}G_{i}(t_{i}^{*})<\left\langle x_{j}G_{j}(t_{j}^{*})\right\rangle . \end{aligned}$$

By the definition of the average operator (2), this is always true in a heterogeneous habitat, and thus $t_{i}^{*}$ always increases with $x_{i}$. Homogeneous habitats, for which $p_{i}=1$ and $\langle x_{j}G_{j}(t_{j}^{*})\rangle =x_{i}G_{i}(t_{i}^{*})$, correspond to the knife-edge case of equality in Eq. (24), so that $dt^{*}/dx=0$ (Eq. 11).

This is illustrated, in the context of function (21) and a three patch-type habitat, in Fig. 2c. Only the limit case of homogeneity (dot on the far right) yields invariance. In all other contexts, $t_{i}^{*}$ increases with $x_{i}$. We also observe in the figure that the smaller $p_{i}$, the steeper the increase of $t_{i}^{*}$ with $x_{i}$ , and that the higher $x_{i}$ (i.e. the richer the patch-type relative to the average), the shallower the increase of $t_{i}^{*}$ with $x_{i}$. Both are illustrations of Proposition 2. Last, we observe that an increase in $p_{i}$ increases (decreases) $t_{i}^{*}$ if it decreases (increases) the overall habitat quality, which illustrates the result (20) obtained in Sect. 5.2

If we consider the average optimal residence time in a habitat, invariance to $x_{i}$ is again not observed in heterogeneous habitats (Fig. 2c). If gain functions have identical second time-derivatives at the MVT optimum (an example of this is (21) with one $\lambda$ value; Appendix), we can use (18) to predict the response of $\langle t_{j}^{*}\rangle$ to $x_{i}$. In the context of function (23), $\langle t_{j}^{*}\rangle$ increases with $x_{i}$ when

$$\begin{aligned} \frac{1}{E_{n}^{*}}p_{i}\frac{dG_{i}(t_{i}^{*})}{dt}-\frac{p_{i}F(t_{i}^{*})}{\left\langle F_{j}(x_{j},t_{j}^{*})\right\rangle }>0\Leftrightarrow x_{i}G_{i}(t_{i}^{*})=F_{i}(x_{i},t_{i}^{*})<\left\langle F_{j}(x_{j},t_{j}^{*})\right\rangle . \end{aligned}$$

Hence, average residence time increases (decreases) with patch quality if the manipulated patch-type yields lower (higher) absolute gains than average at the MVT optimum. Invariance only results when the manipulated patch-type yields exactly average absolute gains ($F_{i}(x_{i},t_{i}^{*})=\langle F_{j}(x_{j},t_{j}^{*})\rangle$).

From the fact that $x_{i}$ is a metric of quality and that, as we have just shown $\partial t_{i}^{*}{/}\partial x_{i}\!>\!0$, we have

$$\begin{aligned} \frac{dF_{i}(x_{i},t_{i}^{*}(\mathbf {x}))}{dx_{i}}=\frac{\partial F_{i}\left( x_{i},t_{i}^{*}\right) }{\partial x_{i}}+\frac{\partial F_{i}(x_{i},t_{i}^{*})}{\partial t_{i}}\frac{\partial t_{i}^{*}}{\partial x_{i}}>0. \end{aligned}$$

In addition, $dF_{k}(x_{k},t_{k}^{*}(\mathbf {x}))/dx_{i}<0$ for all $k\in \Omega$ (from Corollary 1 and the fact that $F_{k}$ is increasing in $t_{k}$ at $t_{k}^{*}$) while $dF_{k}(x_{k},t_{k}^{*}(\mathbf {x}))/dx_{i}=0$ for all $k\notin \Omega$. Hence $F_{i}(x_{i},t_{i}^{*})-\langle F_{j}(x_{j},t_{j}^{*})\rangle =(1-p_{i})F_{i}(x_{i},t_{i}^{*})-\sum _{j\ne i}F_{j}(x_{j},t_{j}^{*})$ is an increasing function of $x_{i}$. The invariance point $F_{i}(x_{i},t_{i}^{*})=\langle F_{j}(x_{j},t_{j}^{*})\rangle$ thus represents a maximum of $\langle t_{j}^{*}\rangle$ with respect to $x_{i}$. This is illustrated Fig. 3a^1^. Since, for each patch-type individually, $\langle t_{j}^{*}\rangle$ is maximized when the patch-type yields exactly average gains, we can further conclude that $\langle t_{j}^{*}\rangle$ is globally maximized when all patch-types have the same $x$ value, i.e. in the homogeneous case. Thus, in a heterogeneous habitat, $\langle t_{j}^{*}\rangle$ is smaller than the $t^{*}$ value one would observe in a homogeneous habitat; heterogeneity always decreases the average optimal strategy. This is visible in Fig. 2d.

In the more general case where second time-derivatives do differ at the MVT optimum (an example of this is function (22); Appendix), Theorem 3 implies that these further influence the response of average residence time. This is illustrated in Fig. 3b, in which the maximum of $\langle t_{j}^{*}\rangle$ no longer coincides with $F_{i}(x_{i},t_{i}^{*})=\langle F_{j}(x_{j},t_{j}^{*})\rangle$. In this case, the manipulated patch-type happens to have a gain function less curved than average in the neighborhood of $F_{i}(x_{i},t_{i}^{*})=\langle F_{j}(x_{j},t_{j}^{*})\rangle$ so that, according to Theorem 3, an increase of $\langle t_{j}^{*}\rangle$ is more likely, all else equal. Consistent with this, the maximum of $\langle t_{j}^{*}\rangle$ is shifted toward higher $x_{i}$ values (Fig. 3b).

Last, if several patch-types are simultaneously altered in the habitat together with patch-type $i$ (*i.e.* $x_{l}=x_{l}(x_{i})$), we get from (11), for any $k\in \Omega$:

25 $$\begin{aligned} \frac{dt_{k}^{*}}{dx_{i}}&= {\displaystyle \sum _{l=1}^{s}\frac{\partial t_{k}^{*}}{\partial x_{l}}\frac{dx_{l}}{dx_{i}}}\nonumber \\&= {\displaystyle -E_{n}^{*}\sum _{l=1}^{s}\left( \frac{\partial }{\partial x_{l}}\ln \frac{\partial F_{k}\left( x_{k},t_{k}^{*}\right) }{\partial t_{k}}-\frac{\partial \ln E_{n}^{*}}{\partial x_{l}}\right) \left( \frac{\partial ^{2}F_{k}\left( x_{k},t_{k}^{*}\right) }{\partial t_{k}^{2}}\right) ^{-1}\frac{dx_{l}}{dx_{i}}}\nonumber \\&= -E_{n}^{*}{\displaystyle \left( \frac{\partial }{\partial x_{k}}\ln \frac{\partial F_{k}\left( x_{k},t_{k}^{*}\right) }{\partial t_{k}}\frac{dx_{k}}{dx_{i}}-\frac{d\ln E_{n}^{*}}{dx_{i}}\right) \left( \frac{\partial ^{2}F_{k}\left( x_{k},t_{k}^{*}\right) }{\partial t_{k}^{2}}\right) ^{-1}}\nonumber \\&= -E_{n}^{*}{\displaystyle \left( \frac{1}{E_{n}^{*}}\frac{\partial ^{2}F_{k}\left( x_{k},t_{k}^{*}\right) }{\partial x_{k}\partial t_{k}}\frac{dx_{k}}{dx_{i}}-\frac{d\ln E_{n}^{*}}{dx_{i}}\right) \left( \frac{\partial ^{2}F_{k}\left( x_{k},t_{k}^{*}\right) }{\partial t_{k}^{2}}\right) ^{-1}.} \end{aligned}$$

The first term in the parenthesis can be simplified as above to yield $(dx_{k}/dx_{i})/x_{i}=d\ln x_{k}/dx_{i}$, so that $dt_{k}^{*}/dx_{i}=0$ if and only if

26 $$\begin{aligned} \frac{d\ln x_{k}}{dx_{i}}=\frac{d\ln E_{n}^{*}}{dx_{i}}\Leftrightarrow \frac{d\ln E_{n}^{*}}{d\ln x_{k}}=1. \end{aligned}$$

From (8), this means, in the context of (23):

27 $$\begin{aligned} \frac{d\ln \left\langle F_{j}(x_{j},t_{j}^{*})\right\rangle }{d\ln x_{k}}=1. \end{aligned}$$

Hence, scaling the gains in one patch-type leaves the optimal residence time unchanged if and only if the scaling is identical to that of the average gain in the habitat. A necessary condition for all optimal residence times $t_{k}^{*}$ to be invariant is to have (26) hold for all $k\in \Omega$: all exploited patch-types should thus have their gain functions scaled in exactly the same way, i.e. $d\ln x_{k}/d\ln x_{i}=1$ for all $k\in \Omega$. However, it still remains to be determined whether equality holds in Eq. (26), which also depends on the variation of $x_{k}$ for non-exploited patches. As shown in Appendix, this imposes an additional constraint on non-exploited patches, which, for instance, is satisfied if $F_{l}(x_{l},0)=0$ (as is often assumed). In any case, a sufficient condition for invariance of all residence times is to have all gain functions (even for non-exploited patches) rescaled in exactly the same way. This type of transformation is illustrated in Fig. 2b. Hence, upon scaling the gains in a heterogeneous habitat, one should preserve the habitat heterogeneity (in the sense that the coefficient of variation of $x$ must stay constant over all exploited patches), otherwise invariance is lost.

#### Scaling the time

A different form of scaling invariance was proposed by Charnov and Parker (1995), based on an approximation of function (21). They reported that if parameter $\lambda$ is increased, and travel time is simultaneously reduced (so that the product $\lambda T$ stays constant), then ${\lambda }t^{*}$ appears to be almost invariant under the MVT. This invariance and the underlying constraint on $\lambda T$ are consistent with data on the duration of copulation in dung-flies (Charnov and Parker 1995). In this context, the relevant patch attribute $x$ is $\lambda$ rather than $\mu$ in (21). Intuitively, increasing $\lambda$ corresponds to accelerating time, and thus the kinetics of gain acquisition, which constitutes another natural way to improve patch quality (Parker and Stuart 1976; Ranta et al. 1995). Remark that decreasing $k$ in function (22) has exactly the same accelerating effect. We are thus led to considering the class of gain functions

28 $$\begin{aligned} F_{i}(x_{i},t)=G_{i}(x_{i}t), \end{aligned}$$

for some $x_{i}>0$ and $G_{i}$, together with having travel time inversely proportional to $x_{i}$, i.e. $T_{i}(x_{i})=\tau _{i}/x_{i}$ for some positive $\tau _{i}$. Class (28) includes both (21) and (22), with $x$ taken to be $\lambda$ and $1/k$, respectively.

**Fig. 4:**
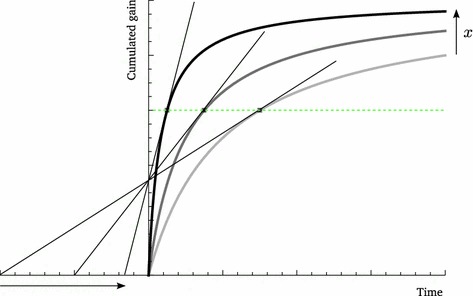
Scaling the time and invariance. Function (22) is used as an illustration of class (28). In a homogeneous habitat, increasing $x$ while decreasing $T$ in the same proportion leaves the product $xt^{*}$ unchanged, as was conjectured by Charnov and Parker (1995). This in turn implies that the absolute gains extracted from a patch are also invariant (*horizontal green line*) (color figure online)

Given that $\partial F_{i}(x_{i},t_{i}^{*})/\partial x_{i}=t_{i}^{*}G_{i}'(x_{i}t_{i}^{*})>0$ and $dT_{i}(x_{i})/dx_{i}<0$, $x_{i}$ is a metric of quality for all $t_{i}^{*}$ (Proposition 1), as was the case for (23). Graphically, just like the earlier form of invariance (Sect. 5.3.1) corresponded to scaling the gain function vertically, the present invariance corresponds to scaling it horizontally, together with travel time. This is illustrated in Fig. 4. Invariance of $x_{i}t_{i}^{*}$ in (28) implies invariance of the absolute gains $F_{i}(x_{i},t_{i}^{*})$, as shown in the figure.

Using our results, we can prove that this invariance property suggested by Charnov and Parker (1995) holds exactly, not only approximately, in homogeneous habitats. However, in heterogeneous habitats, this invariance is again non-generic. Since the approach is the same as above, we will directly consider the case where several patch-types are simultaneously manipulated in the habitat.

From (25), for any exploited patch-type, we can express $dt_{k}^{*}/dx_{i}$ as

$$\begin{aligned} \frac{dt_{k}^{*}}{dx_{i}}&= \left( -G_{k}'(x_{k}t_{k}^{*})-x_{k}t_{k}^{*}G_{k}''(x_{k}t_{k}^{*})\right) \frac{dx_{k}}{dx_{i}}\left( x_{k}^{2}G_{k}''(x_{k}t_{k}^{*})\right) ^{-1}\\&+\,E_{n}^{*}\frac{d\ln E_{n}^{*}}{dx_{i}}\left( x_{k}^{2}G_{k}''(x_{k}t_{k}^{*})\right) ^{-1} \end{aligned}$$

where, as before, $d\ln E_{n}^{*}/dx_{i}$ incorporates the effects of all manipulated patches. Remembering that $E_{n}^{*}=\partial F_{k}\left( x_{k},t_{k}^{*}\right) /\partial t_{k}=x_{k}G_{k}'(x_{k}t_{k}^{*})$, this yields, for all $k\in \Omega$:

$$\begin{aligned} \frac{dt_{k}^{*}}{dx_{i}}=E_{n}^{*}\left( -\frac{1}{x_{k}}\frac{dx_{k}}{dx_{i}}+\frac{d\ln E_{n}^{*}}{dx_{i}}\right) \left( x_{k}^{2}G_{k}^{''}(x_{k}t_{k}^{*})\right) ^{-1}-\frac{t_{k}^{*}}{x_{k}}\frac{dx_{k}}{dx_{i}}. \end{aligned}$$

Noting that $x_{k}t_{k}^{*}$ stays constant if and only if

$$\begin{aligned} \frac{dx_{k}t_{k}^{*}}{dx_{i}}=0\Leftrightarrow \frac{dt_{k}^{*}}{dx_{i}}=-\frac{t_{k}^{*}}{x_{k}}\frac{dx_{k}}{dx_{i}}, \end{aligned}$$

invariance is achieved if and only if

$$\begin{aligned} \frac{d\ln E_{n}^{*}}{dx_{i}}-\frac{1}{x_{k}}\frac{dx_{k}}{dx_{i}}=0, \end{aligned}$$

which leads us to exactly the same condition (26) as for the previous form of scaling invariance.

In the context of (28), it is shown in Appendix that the invariance condition means:

29 $$\begin{aligned} \left\langle \left( T_{j}(x_{j})+t_{j}^{*}\right) \frac{d\ln x_{j}}{d\ln x_{i}}\right\rangle =\left\langle T_{j}(x_{j})+t_{j}^{*}\right\rangle . \end{aligned}$$

We immediately see that it is true in homogeneous habitats, proving the invariance property conjectured by Charnov and Parker (1995), not only for function (21) but for any function in class (28). However, just like the previous form of invariance, this one is non-generic in heterogeneous habitats. In particular, global invariance of the $x_{k}t_{k}^{*}$ results if and only if all exploited patch-types are scaled homogeneously, i.e. $d\ln x_{j}/d\ln x_{i}=1$ for all $j\in \Omega$, and an additional constraint is satisfied for non-exploited patches (Appendix).

### Should one stay longer on better patches?

So far we compared different habitats in the sense that changes in patch attributes caused a change in the overall quality ($E_{n}^{*}$). However, one (intuitive) prediction often attributed to the MVT is that, in a given heterogeneous habitat, optimal residence times should rank in the same order as patch qualities, where quality is intended as having a ’better’ gain function (Parker and Stuart 1976; Kelly 1990; Wajnberg et al. 2000). This was already suggested graphically in the seminal MVT article (Charnov 1976; see also Fig. 1b).

Let us consider that the gain functions all come from varying a parameter $x$ in some function $F$, i.e. $F_{i}(x_{i},t_{i})=F(x_{i},t_{i})$ for all $i$. The classes of gain functions (23)/(28) considered in the previous section, with one function $G$, are instances of this scenario. Since we are only interested in the gain functions, we will assume that travel times do not vary with $x_{i}$. In a given habitat, unexploited patches have null optimal residence times, and all positive optimal residence times are determined from $E_{n}^{*}$, as shown in Fig. 1b. Hence, $x_{i}$ entirely determines $t_{i}^{*}$; for all patch-types $i\in \Omega$, $t_{i}^{*}$ is given by one function of $x_{i}$. If $x_{min}$ is the lowest $x$ value over patch-types in $\Omega$, and $x_{max}$ the highest, greater $x$ values unambiguously represent better patches within the habitat if $x_{i}$ is a metric of quality (Definition 1) for all $x_{i}\in \left( x_{min},x_{max}\right)$. We are interested in determining whether $t_{i}^{*}$ is an increasing function of $x_{i}$, for a given value of $E_{n}^{*}$.

When varying $x_{i}$ for some patch-type $i\in \Omega$, ignoring the variation of habitat quality $E_{n}^{*}$, the change in $t_{i}^{*}$ is obtained from (13) with $\partial \ln E_{n}^{*}/\partial x_{i}$ set to zero. This gives the (total) derivative of $t_{i}^{*}$ as:

30 $$\begin{aligned} \frac{dt_{i}^{*}}{dx_{i}}=-\left( \frac{\partial ^{2}F(x_{i},t_{i}^{*})}{\partial t_{i}^{2}}\right) ^{-1}\frac{\partial ^{2}F(x_{i},t_{i}^{*})}{\partial x_{i}\partial t_{i}}. \end{aligned}$$

**Fig. 5:**
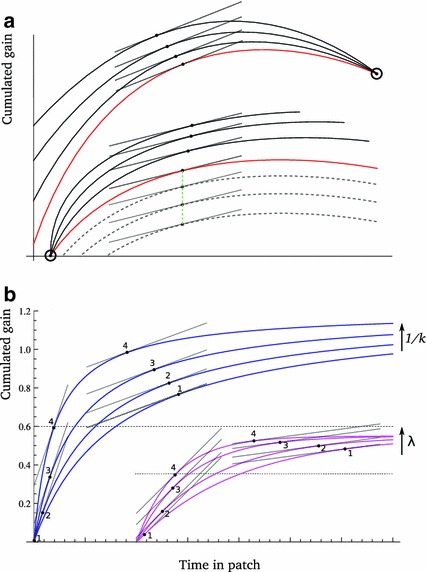
Stay longer on poorer patches? **a** From some gain function (*red*), increasing quality by rotating the function clockwise makes $t_{i}^{*}$ a decreasing function of quality within a habitat (*top*). Rotating the function anti-clockwise makes $t_{i}^{*}$ an increasing function of quality within a habitat (*bottom*). *Dotted gray curves* translating the gain function vertically makes $t_{i}^{*}$ constant over patch-types (*green vertical line*). **b** Two four patch-type habitats are illustrated. In the lowmost (*magenta*) habitat, parameter $\lambda$ was varied in gain function (21). In the topmost (*blue*) habitat, parameter $k$ was varied in gain function (22). In the first case, the four optimal residence times (labeled 1 to 4) increase with patch quality if patches are less than 50 % exploited (*horizontal dashed line*), but decrease if patches are more than 50 % exploited. In the second case, they increase with patch quality if patches are less than 63 % exploited (*horizontal dotted line*), but decrease with quality if patches are more than 63 % exploited. For clarity, the magenta curves were shifted to the right. * Other parameters*: $v_{m}=1.2$, $\mu =0.5$ (color figure online)

The sign of $\partial ^{2}F(x_{i},t_{i}^{*})/\partial x_{i}\partial t_{i}$ is not constrained by $x_{i}$ being a quality metric (Proposition 1), so that $dt_{i}^{*}/dx_{i}$ can have any sign, depending on how the time-derivative of $F$ changes with $x_{i}$. We can immediately conclude from (30) that, in a given habitat, $t_{i}^{*}$ is an increasing function of $x_{i}$ if $\partial ^{2}F(x_{i},t_{i}^{*})/\partial x_{i}\partial t_{i}>0$, and a decreasing function of $x_{i}$ if $\partial ^{2}F(x_{i},t_{i}^{*})/\partial x_{i}\partial t_{i}<0$, for all $x_{i}\in \left( x_{min},x_{max}\right)$. The generic transformation corresponding to these scenarios is rotating the gain functions, with $x_{i}$ representing the angle of rotation. If $x_{i}>0$, $\partial ^{2}F(x_{i},t_{i})/\partial x_{i}\partial t_{i}>0$ for all $t_{i}$, so that $dt_{i}^{*}/dx_{i}>0$: individuals should spend more time on better patches. If $x_{i}<0$, the reverse is true. This is illustrated in Fig. 5a.

Going back to the functions studied in this previous section, it is straightforward to see that varying $x$ in class (23) is an example of the first situation. Indeed, for all $t$ and $x$, we have:

$$\begin{aligned} \frac{\partial ^{2}F(x,t)}{\partial x\partial t}=\frac{dF(t)}{dt}. \end{aligned}$$

As $F$ is increasing in $t$ at any MVT optimum, $dt_{i}^{*}/dx_{i}>0$ (30), for all $x_{i}$. Thus, individuals should indeed spend more time on better patches for this class of gain functions. Figure 2a offered an illustration of this in the case of function (21).

However, even for very similar and natural ways to model patch quality, the MVT can readily yield the opposite prediction that individuals should stay longer on poorer patches. If we consider instead the class of functions (28), for instance the same two functions (21) and (22), the cross derivatives $\partial ^{2}F(x_{i},t_{i}^{*})/\partial x_{i}\partial t_{i}$ are, respectively,

$$\begin{aligned} \mu (1-x_{i}t_{i}^{*})\exp (-x_{i}t_{i}^{*})\,\mathrm{ and }\, v_{m}(1-x_{i}t_{i}^{*})/(1+x_{i}t_{i}^{*})^{3}. \end{aligned}$$

It follows that, in both cases, they are positive if

$$\begin{aligned} t_{i}^{*}<1/x_{i}. \end{aligned}$$

Remembering that parameter $k$ in (22) is the half-saturation constant, i.e. the time it takes to obtain gains $v_{m}/2$, we immediately see that $t_{i}^{*}<1/x_{i}$ if and only if the patch-type is less than half-depleted. Therefore, $t_{i}^{*}$ is an increasing function of $x_{i}$ if all exploited patches are less than half-depleted, but a decreasing function of $x_{i}$ if all are more than half-depleted. Similarly, in the first case, $t_{i}^{*}<1/x_{i}$ implies that the relative exploitation of patches should be no more than $F(x_{i},1/x_{i})/\mu =1-e^{-1}$, which is about 63 %. These predictions are illustrated in Fig. 5b.

Remark that, from Eq.(30), if the time-derivative of $F$ does not vary with $x_{i}$, $dt_{i}^{*}/dx_{i}=0$ and the optimal residence time will be the same on all patch-types, irrespective of their quality. It will thus be the same as $t^{*}$ in a similar homogeneous habitat. The generic transformation corresponding to this situation is varying quality by translating gain functions vertically, i.e. $F(x_{i},t)=F(t)+x_{i}$ (Fig. 5a). This can describe instant rewards obtained upon entering and/or leaving patches, such as the reward of biting for cheaters in cleaning mutualisms (Bshary et al. 2008). Therefore, just like scaling the gain functions vertically (functions (23) in the previous section) was an identity transformation in homogeneous habitats, translating the gain functions vertically is an identity transformation in heterogeneous habitats, as optimal residence time is invariant to heterogeneity.

Finally, Eq.(30) reveals an affinity between the sign of variation of optimal residence times with quality (Theorems 1–3) and the ordering of optimal residence times with respect to quality in a habitat. Indeed, in both cases, the key element is the sign of $\partial ^{2}F(x_{i},t_{i}^{*})/\partial x_{i}\partial t_{i}$. If it is negative for all $x$, optimal residence times are lower on better patches, while, from Theorems 1-3, $t_{i\in \Omega }^{*}$ and $\langle t_{j}^{*}\rangle$ all decrease with quality. If it is positive for all $x$, optimal residence times are longer on better patches, while $t_{i\in \Omega }^{*}$ and $\langle t_{j}^{*}\rangle$ might increase with quality. This shows that the condition for optimal strategies to decrease with quality is similar to, but less stringent, than the condition for residence times to rank in reverse order of patch qualities within habitats. As an example, in Fig. 5b, while we can be sure that $t^{*}$ would decrease with $x$ when $t_{i}^{*}$ is a decreasing function of $x_{i}$ within habitats (i.e. when patches are sufficiently depleted), the fact that $t_{i}^{*}$ is an increasing function of $x_{i}$ when patches are little depleted does not guarantee that $t^{*}$ would increase with $x$ (actually, using the type of construct shown in Fig. 1a, one can visualize that $t^{*}$ always decreases with $x$, as an application of Theorem 1 would confirm).

## Conclusions and perspectives

The Marginal Value Theorem (MVT; Charnov 1976) offers a fairly general theoretical connection between the attributes of patchy habitats and optimal foraging strategies (Stephens and Krebs 1986). However, as it only provides an implicit definition of optimal strategies, general predictions on the consequences of habitat alterations have remained elusive, with strong reliance on graphical arguments. Here we have reanalysed the MVT in order to provide such general predictions on how optimal strategies should vary with habitat characteristics. We found that some existing predictions were indeed robust: we confirmed the effect of increasing travel time in a more general setting (Sect. 5.1) and proved an invariance property conjectured by Charnov and Parker (1995)(Sect. 5.3). However, several predictions sometimes attributed to the MVT did not prove robust.

First, there is no general trend between optimal residence times and quality: the former can increase or decrease with quality, depending on the exact way gain functions are transformed. We have provided general guidelines regarding what sort of transformations would yield one or the other outcome (Theorems 1 and 2). The crucial point is how the time-derivative of the gain function varies with quality: only if it increases sufficiently can optimal residence time go up with quality. Any habitat alteration that does not make gain functions steeper (including changing the relative abundances of patch-types; Sect. 5.2) invariably yields a decrease of optimal residence time with quality. Second, even within a given habitat, optimal residence times do not necessarily rank in the same order as patch qualities, i.e. one should not always spend more time on better patches: the contrary can, counterintuitively, be optimal. The conditions for this are similar, but more stringent, than those required to observe a lower patch residence time following increased patch quality (Sect. 5.4). Last, the scaling invariances of optimal strategies that were proposed for homogenous habitats(e.g. Parker and Stuart 1976; Charnov and Parker 1995; Ranta et al. 1995) have been shown to be non-generic in heterogeneous habitats (Sect. 5.3). Interestingly, however, we obtained a prediction that the average rate of movement should always be higher in a heterogeneous habitat than in a homogeneous habitat, in the often-considered scenario where patch quality corresponds to a vertical scaling of the gain function.

Our results help better understand the consequences of habitat heterogeneity. All else equal, optimal residence time is more likely to increase with patch quality in a heterogeneous, rather than homogeneous, habitat. This is especially true if the focal patch of interest is rare in its habitat, and is poorer than the average patch (Proposition 2). This indicates that predicting the effect of increasing patch-quality, in experimental settings where the whole habitat context is not known, is hazardous. The non-genericity of the above-mentioned invariances was a manifestation of this. However, a strong prediction emerges: increasing the quality of some patch-types always decrease the optimal residence time on all other exploited patches (Corollary 1). We also provided a comparison between the average behavior of heterogeneous habitats and that of an “average homogeneous habitat”. We have shown that the two behave similarly only if there is no heterogeneity in the curvature of gain functions (at the optimal residence times). Otherwise, a given change in average habitat characteristics might elicit contrasted responses of the average residence time, depending on which patches are altered (Theorem 3). As a consequence, some patches may have disproportionately stronger impact than one would expect based on mean-field considerations, qualifying as keystones (Mouquet et al. 2013). In practice, determining if we are in this sort of situation necessitates estimating curvatures of gain functions respect to time, and predictions involve the harmonic mean of curvatures, which is the appropriate mean in this context. These are much more demanding tasks from a statistical perspective, adding to the challenge of prediction in heterogeneous habitats, compared to homogeneous habitats.

The general results we obtained for heterogeneous habitats pave the way for more applications of the MVT at the level of whole habitats, whereas it is traditionally used at the level of specific patches (Stephens and Krebs 1986). Experimental microcosms appear particularly well-suited to test our predictions (e.g. Friedenberg 2003). These new developments on the MVT can be applied to specific gain functions, as we did in the applications, to obtain precise predictions tailored to particular systems or scenarios. They also provide a framework to assess, in all generality, the robustness of other predictions that have been proposed from graphical arguments and tested experimentally, for instance that varying travel times should have a stronger impact on residence time in richer habitats (Muratori et al. 2008).

## Second time-derivatives of functions (21) and (22)

With $x\triangleq \mu$, the second time-derivative of function (21) is

$$\begin{aligned} \frac{\partial ^{2}F_{i}(x_{i},t_{i}^{*})}{\partial t^{2}}=-\exp (-\lambda t_{i}^{*})\lambda ^{2}x_{i}=-\lambda \frac{\partial F_{i}(x_{i},t_{i}^{*})}{\partial t}=-\lambda E_{n}^{*}. \end{aligned}$$

If $\mu$ is the only quantity that varies among patches, all second time-derivatives are equal at a MVT optimum. This is not the case if $\lambda$ also varies, however. Interestingly, the former property is lost when using more tractable approximations of (21) (e.g. Parker and Stuart 1976).

With $x\triangleq v_{m}$, the second time-derivative of function (22) is

$$\begin{aligned} -2kv_{mi}/(t_{i}^{*}+k)^{3}=-2/(t_{i}^{*}+k)E_{n}^{*}. \end{aligned}$$

Since the $t_{i}^{*}$ are not the same in all patch-types, second-time derivatives differ across patch-types at a MVT optimum.

## Scaling the gains (function (23)) and the invariance of all optimal residence times

Using Eq. (26) in the case $k=i$, we get

31 $$\begin{aligned} \frac{1}{x_{i}}=\frac{d\ln E_{n}^{*}}{dx_{i}}. \end{aligned}$$

From (8), and as that travel times do not vary, we have

$$\begin{aligned} \frac{d\ln E_{n}^{*}}{dx_{i}}=\sum _{l=1}^{s}\frac{\partial \ln \left\langle F_{j}(x_{j},t_{j}^{*})\right\rangle }{\partial x_{l}}\frac{dx_{l}}{dx_{i}}. \end{aligned}$$

Invariance of all residence times necessitates, for all $l\in \Omega$, $d\ln x_{l}/d\ln x_{i}=1\Leftrightarrow dx_{l}/dx_{i}=x_{l}/x_{i}$, so that we can write:

$$\begin{aligned} \frac{d\ln E_{n}^{*}}{dx_{i}}=\sum _{l\in \Omega }\frac{p_{l}}{\left\langle F_{j}(x_{j},t_{j}^{*})\right\rangle }\frac{\partial F_{l}(x_{l},t_{l}^{*})}{\partial x_{l}}\frac{x_{l}}{x_{i}}+\sum _{l\notin \Omega }\frac{p_{l}}{\left\langle F_{j}(x_{j},t_{j}^{*})\right\rangle }\frac{\partial F_{l}(x_{l},t_{l}^{*})}{\partial x_{l}}\frac{dx_{l}}{dx_{i}}. \end{aligned}$$

Since $\partial F_{l}(x_{l},t_{l}^{*})/\partial x_{l}=G_{l}(t_{l}^{*})=F_{l}(x_{l},t_{l}^{*})/x_{l}$, this yields

$$\begin{aligned} \frac{d\ln E_{n}^{*}}{dx_{i}}=\frac{1}{x_{i}\left\langle F_{j}(x_{j},t_{j}^{*})\right\rangle }\left[ \sum _{l\in \Omega }p_{l}F_{l}(x_{l},t_{l}^{*})+\sum _{l\notin \Omega }p_{l}\frac{F_{l}(x_{l},0)}{x_{l}}x_{i}\frac{dx_{l}}{dx_{i}}\right] , \end{aligned}$$

and finally, remembering the definition of the average (2):

$$\begin{aligned} \frac{d\ln E_{n}^{*}}{dx_{i}}=\frac{1}{x_{i}\left\langle F_{j}(x_{j},t_{j}^{*})\right\rangle }\left[ \left\langle F_{j}(x_{j},t_{j}^{*})\right\rangle +\sum _{l\notin \Omega }p_{l}F_{l}(x_{l},0)\left( \frac{x_{i}}{x_{l}}\frac{dx_{l}}{dx_{i}}-1\right) \right] . \end{aligned}$$

When $d\ln x_{l}/d\ln x_{i}=1$ for all $l\in \Omega$, Eq. (31) is thus satisfied if and only if

$$\begin{aligned} \sum _{l\not \in \Omega }p_{l}F_{l}(x_{l},0)\left( \frac{d\ln x_{l}}{d\ln x_{i}}-1\right) =0. \end{aligned}$$

Note that this condition is trivially verified if $F_{l}(x_{l},0)=0$, as is often assumed in this context (functions (21) and (22) are examples). Another sufficient condition for it to be verified is having all non-exploited patch-types satisfy $d\ln x_{l}/d\ln x_{i}=1$ as well.

## Scaling the time (function (28)) and the invariance of all optimal residence times

From (8) we have

$$\begin{aligned} \frac{d\ln E_{n}^{*}}{dx_{i}}&= \sum _{l=1}^{s}\left( \frac{\partial \ln \left\langle F_{j}(x_{j},t_{j}^{*})\right\rangle }{\partial x_{l}}-\frac{d\ln \left\langle T_{j}(x_{j})\right\rangle }{dx_{l}}\frac{\left\langle T_{j}(x_{j})\right\rangle }{\left\langle T_{j}(x_{j})+t_{j}^{*}\right\rangle }\right) \frac{dx_{l}}{dx_{i}}\\ \Leftrightarrow \frac{d\ln E_{n}^{*}}{dx_{i}}&= \sum _{l=1}^{s}p_{l}\left( \frac{1}{\left\langle F_{j}(x_{j},t_{j}^{*})\right\rangle }\frac{\partial F_{l}(x_{l},t_{l}^{*})}{\partial x_{l}}-\frac{dT_{l}(x_{l})}{dx_{l}}\frac{1}{\left\langle T_{j}(x_{j})+t_{j}^{*}\right\rangle }\right) \frac{dx_{l}}{dx_{i}}. \end{aligned}$$

Replacing $\langle F_{j}(x_{j},t_{j}^{*})\rangle$ with $E_{n}^{*}\langle T_{j}(x_{j})+t_{j}^{*}\rangle$, we get

$$\begin{aligned} \frac{d\ln E_{n}^{*}}{dx_{i}}=\frac{1}{\left\langle T_{j}(x_{j})+t_{j}^{*}\right\rangle }\sum _{l=1}^{s}p_{l}\left( \frac{1}{E_{n}^{*}}\frac{\partial F_{l}(x_{l},t_{l}^{*})}{\partial x_{l}}-\frac{dT_{l}(x_{l})}{dx_{l}}\right) \frac{dx_{l}}{dx_{i}}. \end{aligned}$$

Since $\partial F_{l}(x_{l},t_{l}^{*})/\partial x_{l}=t_{l}^{*}G'(x_{l}t_{l}^{*})=(t_{l}^{*}/x_{l})\partial F_{l}(x_{l},t_{l}^{*})/\partial t_{l}=(t_{l}^{*}/x_{l})E_{n}^{*}$, this yields

$$\begin{aligned} \frac{d\ln E_{n}^{*}}{dx_{i}}=\frac{1}{\left\langle T_{j}(x_{j})+t_{j}^{*}\right\rangle }\sum _{l=1}^{s}p_{l}\left( \frac{t_{l}^{*}}{x_{l}}-\frac{dT_{l}(x_{l})}{dx_{l}}\right) \frac{dx_{l}}{dx_{i}}, \end{aligned}$$

and, remembering that $dT_{l}(x_{l})/dx_{l}=-\tau _{l}/x_{l}^{2}=-T_{l}(x_{l})/x_{l}$,

32 $$\begin{aligned} \frac{d\ln E_{n}^{*}}{dx_{i}}=\frac{1}{\left\langle T_{j}(x_{j})+t_{j}^{*}\right\rangle }\sum _{l=1}^{s}p_{l}\left( \frac{t_{l}^{*}}{x_{l}}+\frac{T_{l}(x_{l})}{x_{l}}\right) \frac{dx_{l}}{dx_{i}}. \end{aligned}$$

The invariance condition (31) thus means

$$\begin{aligned} \left\langle \left( T_{j}(x_{j})+t_{j}^{*}\right) \frac{d\ln x_{j}}{d\ln x_{i}}\right\rangle =\left\langle T_{j}(x_{j})+t_{j}^{*}\right\rangle , \end{aligned}$$

which is (29) in the main text.

Now, separating exploiting and non-exploited patches, and since $t_{l}^{*}=0$ for $l\not \in \Omega$, (32) yields

$$\begin{aligned} \frac{d\ln E_{n}^{*}}{dx_{i}}=\frac{1}{\left\langle T_{j}(x_{j})+t_{j}^{*}\right\rangle }\left[ \sum _{l \in \Omega }p_{l}\left( \frac{t_{l}^{*}}{x_{l}}+\frac{T_{l}(x_{l})}{x_{l}}\right) \frac{dx_{l}}{dx_{i}}+\sum _{l\not \in \Omega }p_{l}\frac{T_{l}(x_{l})}{x_{l}}\frac{dx_{l}}{dx_{i}}\right] . \end{aligned}$$

Since invariance of all residence times necessitates, for all $l\in \Omega$, $d\ln x_{l}/d\ln x_{i}=1\Leftrightarrow dx_{l}/dx_{i}=x_{l}/x_{i}$, we get

$$\begin{aligned} \frac{d\ln E_{n}^{*}}{dx_{i}}=\frac{1}{x_{i}\left\langle T_{j}(x_{j})+t_{j}^{*}\right\rangle }\left[ \sum _{l\in \Omega }p_{l}\left( t_{l}^{*}+T_{l}(x_{l})\right) +\sum _{l\not \in \Omega }p_{l}T_{l}(x_{l})\frac{x_{i}}{x_{l}}\frac{dx_{l}}{dx_{i}}\right] , \end{aligned}$$

and, remembering the definition of the average (2), we can express this as

$$\begin{aligned} \frac{d\ln E_{n}^{*}}{dx_{i}}=\frac{1}{x_{i}\left\langle T_{j}(x_{j})+t_{j}^{*}\right\rangle }\left[ \left\langle T_{j}(x_{j})+t_{j}^{*}\right\rangle +\sum _{l\not \in \Omega }p_{l}T_{l}(x_{l})\left( \frac{x_{i}}{x_{l}}\frac{dx_{l}}{dx_{i}}-1\right) \right] . \end{aligned}$$

Thus, the invariance condition (31) is satisfied if and only if

$$\begin{aligned} \sum _{l\not \in \Omega }p_{l}T_{l}(x_{l})\left( \frac{d\ln x_{l}}{d\ln x_{i}}-1\right) =0. \end{aligned}$$

As for the previous form of invariance, a sufficient condition is to have all non-exploited patch-types satisfy $d\ln x_{l}/d\ln x_{i}=1$ as well. However, in this case, the classic assumption $F_{l}(x_{l},0)=0$ does not suffice to satisfy the constraint on unexploited patch-types. The analogous assumption would be $T_{l}(x_{l})=0$ for all $l\notin \Omega$, which is not feasible.

## Footnotes

1 Remark that with function (21), the switch in the sign of $F_{i}(x_{i},t_{i}^{*})-\langle F_{j}(x_{j},t_{j}^{*})\rangle$ coincides with that of $x_{i}-\langle x_{j}\rangle$, but this cannot be expected to be general.

## References

[CR1] Alonso JA, Alonso JC, Carrascal LM, Munoz-Pulido R (1994). Flock size and foraging decisions in central place foraging white storks, *Ciconia ciconia*. Behaviour.

[CR2] Astrom M, Lundberg P, Danell K (1990). Partial prey consumption by browsers: trees as patches. J Anim Ecol.

[CR3] Baker RR (1978). The evolutionary ecology of animal migration.

[CR4] Belisle M (2005). Measuring landscape connectivity: the challenge of behavioral landscape ecology. Ecology.

[CR5] Bonser R, Wright PJ, Bament S, Chukwu UO (1998). Optimal patch use by foraging workers of *lasius fuliginosus*, *l. niger* and *Myrmica ruginodis*. Ecol Entomol.

[CR6] Bowler DE, Benton TG (2005). Causes and consequences of animal dispersal strategies: relating individual behaviour to spatial dynamics. Biol Rev.

[CR7] Brown JS (1988). Patch use as an indicator of habitat preference, predation risk, and competition. Behav Ecol Sociobiol.

[CR8] Bshary R, Grutter AS, Willener AST, Leimar O (2008). Pairs of cooperating cleaner fish provide better service quality than singletons. Nature.

[CR9] Bull JJ, Pfennig DW, Wang I-N (2004). Genetic details, optimization and phage life histories. Trends Ecol Evol.

[CR10] Charnov EL (1976). Optimal foraging the marginal value theorem. Theoret Popul Biol.

[CR11] Charnov EL, Parker GA (1995). Dimensionless invariants from foraging theory’s marginal value theorem. Proc Natl Acad Sci.

[CR12] Corley JC, Villacide JM, van Nouhuys S (2010). Patch time allocation by a parasitoid: the influence of con-specifics, host abundance and distance to the patch. J Insect Behav.

[CR13] Danchin É, Giraldeau LA, Cézilly F (2008). Behavioural ecology.

[CR14] Friedenberg NA (2003). Experimental evolution of dispersal in spatiotemporally variable microcosms. Ecol Lett.

[CR15] Hayden BY, Pearson JM, Platt ML (2011). Neuronal basis of sequential foraging decisions in a patchy environment. Nat Neurosci.

[CR16] Kelly CK (1990). Plant foraging: a marginal value model and coiling response in *Cuscuta subinclusa*. Ecology.

[CR17] Livoreil B, Giraldeau L (1997). Patch departure decisions by spice finches foraging singly or in groups. Anim Behav.

[CR18] Lundberg P, Danell K (1990) Functional response of browsers: tree exploitation by moose. Oikos

[CR19] McNair JN (1982). Optimal giving-up times and the marginal value theorem. Am Nat.

[CR20] Mouquet N, Gravel D, Massol F, Calcagno V (2013). Extending the concept of keystone species to communities and ecosystems. Ecol Lett.

[CR21] Muratori F, Boivin G, Hance T (2008). The impact of patch encounter rate on patch residence time of female parasitoids increases with patch quality. Ecol Entomol.

[CR22] Nolet BA, Klaassen M (2009). Retrodicting patch use by foraging swans in a heterogeneous environment using a set of functional responses. Oikos.

[CR23] Nonacs P (2001). State dependent behavior and the marginal value theorem. Behav Ecol.

[CR24] Parker GA, Stuart RA (1976). Animal behavior as a strategy optimizer: evolution of resource assessment strategies and optimal emigration thresholds. Am Nat.

[CR25] Poethke HJ, Hovestadt T (2002). Evolution of density-and patch-size-dependent dispersal rates. Proc R Soc Lond Ser B Biol Sci.

[CR26] Ranta E, Rita H, Peuhkuri N (1995). Patch exploitation, group foraging, and unequal competitors. Behav Ecol.

[CR27] Riechert SE, Gillespie RG (1986) Habitat choice and utilization in web-building spiders. Webs, Behavior and Evolution, Spiders

[CR28] Rijnsdorp AD, Poos JJ, Quirijns FJ (2011). Spatial dimension and exploitation dynamics of local fishing grounds by fishers targeting several flatfish species. Can J Fish Aquat Sci.

[CR29] Rita H, Ranta E, Peuhkuri N (1997). Group foraging, patch exploitation time and the finder’s advantage. Behav Ecol Sociobiol.

[CR30] Sih A (1980). Optimal foraging: partial consumption of prey. Am Nat.

[CR31] Stephens DW, Dunbar SR (1993). Dimensional analysis in behavioral ecology. Behav Ecol.

[CR32] Stephens DW, Krebs JR (1986). Foraging theory.

[CR33] Tenhumberg B, Keller MA, Possingham HP, Tyre AJ (2001). Optimal patch-leaving behaviour: a case study using the parasitoid *Cotesia rubecula*. J Anim Ecol.

[CR34] Thompson D, Fedak MA (2001). How long should a dive last? A simple model of foraging decisions by breath-hold divers in a patchy environment. Anim Behav.

[CR35] Wajnberg E, Fauvergue X, Pons O (2000). Patch leaving decision rules and the marginal value theorem: an experimental analysis and a simulation model. Behav Ecol.

[CR36] Wajnberg E, Bernhard P, Hamelin F, Boivin G (2006). Optimal patch time allocation for time-limited foragers. Behav Ecol Sociobiol.

[CR37] Wilson K, Lessells CM (1994). Evolution of clutch size in insects. i. A review of static optimality models. J Evol Biol.

